# Preservative

**Published:** 1879-09

**Authors:** 


### PRESERVATIVE.

A ham well packed in pulverized charcoal, after the usual smoking, will keep for years. Butter put into clean pots and well surrounded with charcoal will keep good for twelve months; this is the antiseptic quality of charcoal, arising from the fact that each atom has the capacity of absorbing a thousand times its bulk of deleterious gases, and thus keeps what it surrounds in perfect purity.

